# Clinical Course of Infection and Cross-Species Detection of Equine Parvovirus-Hepatitis

**DOI:** 10.3390/v13081454

**Published:** 2021-07-26

**Authors:** Birthe Reinecke, Mara Klöhn, Yannick Brüggemann, Volker Kinast, Daniel Todt, Alexander Stang, Marcha Badenhorst, Katja Koeppel, Alan Guthrie, Ursula Groner, Christina Puff, Madeleine de le Roi, Wolfgang Baumgärtner, Jessika-M. V. Cavalleri, Eike Steinmann

**Affiliations:** 1 Institute of Experimental Virology, TWINCORE, a Joint Venture between Hannover Medical School and Helmholtz Centre for Infection Research, 30625 Hannover, Germany; birthe.reinecke@twincore.de; 2 Department of Molecular and Medical Virology, Ruhr-University Bochum, 44801 Bochum, Germany; Mara.Kloehn@ruhr-uni-bochum.de (M.K.); Yannick.Brueggemann@ruhr-uni-bochum.de (Y.B.); Volker.Kinast@rub.de (V.K.); Daniel.Todt@ruhr-uni-bochum.de (D.T.); Alexander.Stang@ruhr-uni-bochum.de (A.S.); 3 European Virus Bioinformatics Center (EVBC), 07743 Jena, Germany; 4 Department for Companion Animals and Horses, University of Veterinary Medicine, 1210 Vienna, Austria; marcha.badenhorst@vetmeduni.ac.at (M.B.); Jessika.Cavalleri@vetmeduni.ac.at (J.-M.V.C.); 5 Department of Companion Animal Clinical Studies, Faculty of Veterinary Science, University of Pretoria, Onderstepoort, Pretoria 0110, South Africa; 6 Department of Production Animal Studies, Faculty of Veterinary Science, University of Pretoria, Onderstepoort, Pretoria 0110, South Africa; katja.koeppel@up.ac.za; 7 Centre for Veterinary Wildlife Studies, Faculty of Veterinary Sciences, University of Pretoria, Onderstepoort, Pretoria 0110, South Africa; 8 Equine Research Centre, Faculty of Veterinary Science, University of Pretoria, Onderstepoort, Pretoria 0110, South Africa; alan.guthrie@up.ac.za; 9 Economic Cooperative of German Veterinarians e.G. (WDT), 27318 Hoyerhagen, Germany; u.groner@gmx.de; 10 Department of Pathology, University of Veterinary Medicine Hannover, 30559 Hannover, Germany; Christina.Puff@tiho-hannover.de (C.P.); Madeleine.De.le.Roi@tiho-hannover.de (M.d.l.R.); Wolfgang.Baumgaertner@tiho-hannover.de (W.B.); 11 Clinic for Horses, University of Veterinary Medicine Hannover, Foundation, 30559 Hannover, Germany

**Keywords:** equine parvovirus hepatitis, hepatopathy, phylogeny, persistent viremia, Theiler’s disease

## Abstract

Since its first discovery by Arnold Theiler in 1918, serum hepatitis also known as Theiler’s disease has been reported worldwide, causing idiopathic acute hepatitis and liver failure in horses. Recent studies have suggested a novel parvovirus, named equine parvovirus hepatitis (EqPV-H), to be associated with Theiler’s disease. Despite the severity and potential fatality of EqPV-H infection, little is known about the possibility of developing chronic infections and putative cross-species infection of equine sister species. In the present longitudinal study, we employed qPCR analysis, serology, and biochemical testing as well as pathology examination of liver biopsies and sequence analysis to investigate potential chronic EqPV-H infection in an isolated study cohort of in total 124 horses from Germany over five years (2013–2018). Importantly, our data suggest that EqPV-H viremia can become chronic in infected horses that do not show biochemical and pathological signs of liver disease. Phylogenetic analysis by maximum likelihood model also confirms high sequence similarity and nucleotide conservation of the multidomain nuclear phosphoprotein NS1 sequences from equine serum samples collected between 2013–2018. Moreover, by examining human, zebra, and donkey sera for the presence of EqPV-H DNA and VP1 capsid protein antibodies, we found evidence for cross-species infection in donkey, but not to human and zebra. In conclusion, this study provides proof for the occurrence of persistent EqPV-H infection in asymptomatic horses and cross-species EqPV-H detection in donkeys.

## 1. Introduction

Serum hepatitis also known as Theiler’s disease (TD) is the most common cause of acute and potentially life-threatening viral hepatitis in horses. The disease was first described by Arnold Theiler in 1918, who observed acute liver atrophy and diffuse hepatic necrosis following the administration of a combination of live virus and convalescent equine antiserum in horses from South Africa [1]. Since then, outbreaks of TD have been described worldwide in conjunction with a vast variety of equine-derived blood products including tetanus antitoxin, botulinum antitoxin, antiserum against *Streptococcus equi* as well as pregnant mare’s serum, and equine plasma [1,2,3,4,5]. Incidence rates of fulminant hepatitis in horses receiving an equine-derived product have been reported to vary between 1.4% and 18% [1,6].

In the past, three flaviviruses including equine hepacivirus, (EqHV), *Pegivirus D*, and *Pegivirus E* have been suggested to be associated with TD [5,7,8,9,10]. However, only EqHV has displayed signs of being hepatotropic in horses [11]. Interestingly, Divers et al. [2] recently identified a novel parvovirus designated as equine parvovirus-hepatitis (EqPV-H) in the serum and liver of a horse that died in Nebraska (USA) 65 days after prophylactic treatment with equine-origin tetanus antitoxin (TAT). Subsequent infection experiments in two healthy mares inoculated with commercial TAT that were qPCR positive for EqPV-H DNA confirmed EqPV-H as the pathogenic agent of idiopathic acute hepatitis and liver failure in horses a.k.a. Theiler’s disease [2,12,13].

Since then, a high prevalence of EqPV-H genomes has been identified in commercially available horse serum samples from the USA, Canada, New Zealand, Italy, and Germany indicating a worldwide distribution of EqPV-H [14]. Apart from iatrogenic transmission, incidences of EqPV-H infections in horses without exposure to biological equine serum products have been described. For instance, Tomlinson et al. reported in 2019, that EqPV-H DNA was present in 9 out of 10 TD cases that did not receive biological equine serum products and noted that 54% of tested in-contact horses were EqPV-H positive [6]. Furthermore, numerous outbreaks of TD in association with EqPV-H have been described in various study cohorts from USA [2,6], Canada [15], Europe [16], and China [17,18].

EqPV-H is a member of the *Parvovirinae* subfamily comprising of small, non-enveloped DNA viruses that are known to infect a wide range of hosts including humans, domestic, and wild animals [19,20,21]. Based on genome organization and genetic relatedness to known parvoviruses, EqPV-H has further been classified into the *Copiparvovirus* genus [2]. In addition, two large open reading frames (ORFs) have been predicted in the complete 5308 nucleotides (nt) genome of EqPV-H which encodes for at least two major gene complexes, the non-structural protein (NS1, multidomain nuclear phosphoprotein) and capsid protein (VP1) [2].

Even though, EqPV-H has been the most common cause of life-threatening viral hepatitis in horses, the possibility of EqPV-H persistence and cross-species EqPV-H detection in equine sister species remains poorly investigated. By monitoring the prevalence of EqPV-H DNA, anti-VP1 antibodies, serum biochemical parameters, pathology, and viral sequence phylogeny in horse serum samples of an isolated study cohort in Germany taken between 2013 and 2018, we first investigated the possibility of persistent EqPV-H infections over a five-year period. To assess putative cross-species infections, human, zebra, and donkey sera were analyzed for the presence of anti-EqPV-H antibodies and DNA using luciferase immunoprecipitation systems (LIPS) assay and qPCR, respectively. Our results suggest that EqPV-H infection can become persistent leading to chronic EqPV-H viremia in subclinical horses. Furthermore, this study provides evidence of cross-species infections of EqPV-H in donkeys.

## 2. Materials and Methods

### 2.1. Serum Sample Collection

Serum samples of in total 124 horses were collected for diagnostic purposes from an isolated cohort in Germany between 2013 and 2018. All serum samples were collected by a veterinarian and stored at −80 °C. Due to euthanization and introduction of new horses, only a subgroup of 45 horses were available at every sampling date. A total of 318 human serum samples (147 samples from human without horse contact, 171 samples from human with occupational horse contact [11]) and 494 donkey serum samples originated from Germany (78), Italy (317), and Bulgaria (99) [22] were donated by volunteers and collected from across Europe, respectively. Archived Burchell’s (65 samples) and Cape mountain (231 samples) zebra sera, collected between 2009 and 2017 across South Africa, were acquired from a biobank. Zebra serum samples were chosen based on availability of serum and compliance with regulations for export to Europe. Samples were shipped to Germany on dry ice and stored at −80 °C until further processing.

### 2.2. Detection of EqPV-H DNA

We used the QIAamp DNA Blood Mini Kit (Cat. No. 51106, Qiagen, Hilden, Germany) to extract viral DNA from serum and liver biopsy samples according to the manufacturer’s recommendations and stored eluate at −20 °C until further analysis. For tissue specimens AL buffer was replaced with 180 µL ATL buffer (Cat. No.939011, Qiagen) supplemented with 20 µL Proteinase K (Cat. No. 19131, Qiagen). A probe-based quantitative polymerase chain reaction (qPCR) was performed using primers and probes previously designed by Dr. Amit Kapoor (Table 1). In brief, qPCR was performed using the QuantiTect Probe PCR Kit (Cat. No. 204343, Qiagen) in a total volume of 30 µL containing 2 µL of purified DNA, 10 µL of 2× Master Mix, 1 µL of parvovirus Taqman probe and 10 pmol of each primer. The qPCR profile was the following: 50 °C for 2 min, 95 °C for 10 min, 44 cycles of 95 °C for 30 s, and 58 °C for 1 min. A plasmid containing the EqPV-H VP1 sequence was serially diluted to quantify the amount of EqPV-H DNA in each sample. qPCR measurements were performed using the LightCycler 480 real-time PCR system (Roche, Mannheim, Germany). Limit of detection (LOD) and limit of quantification (LOQ) was 250 DNA copies/mL and 2500 DNA copies/mL for serum specimens, respectively. EqPV-H positive serum samples below LOQ were additionally checked for virus specific amplification on agarose gel.

### 2.3. Detection of Anti-EqPV-H Antibodies

Anti-EqPV-H-VP1 antibodies in samples were measured using the previously described LIPS assay [8,23]. EqPV-H VP1 antigen was produced and used in the LIPS. Relative light units (RLU) were determined using a plate luminometer (LB 960 XS3; Berthold, Bad Wildbad, Germany). Samples were tested in technical duplicates. To calculate sensitivity, the mean RLU plus three standard deviations (SD) of an EqPV-H negative horse serum was defined as threshold. A commercial horse serum, which was previously tested positive for the presence of EqPV-H DNA, was used as positive control in all assays.

### 2.4. Histology

Liver biopsies and corresponding serum samples were obtained from three horses (6, 11, 72) in 2019. Prior to percutaneous liver biopsy, horses were sedated with detomidine IV (0.006 mg/kg, Cepesedan) and butorphanol IV (0.012 mg/kg, Alvegesic). After clipping and aseptic preparation of the biopsy site, the insertion site was injected with 2% lidocaine subcutaneously and infiltrating intercostal muscles after locating the biopsy needle insertion site by ultrasonography. Biopsies were performed using a semiautomated biopsy device and single use biopsy needles. Samples were fixed in 10% neutrally buffered formalin, routinely embedded in paraffin wax, sectioned at 3 µm and stained with hematoxylin and eosin (HE).

### 2.5. Measurement of Liver Specific Biochemical Analytes

Serum samples of 24 horses in 2015 and 2018 were tested for biochemical analytes indicative for hepatic disease. Samples were chosen based on available volume of serum samples and on EqPV-H DNA status in 2015 and 2018. Samples were submitted for the following biochemical tests: albumin, total bilirubin, bile acids, glutamate dehydrogenase (GLDH), gamma-glutamyl transferase (GGT) and aspartate aminotransferase (AST). Tests were performed at the central laboratory of the small animal hospital (Justus-Liebig-Universität Gießen) using a Hitachi Cobas 6000 c501 (Roche Diagnostics, Laval, QC, Canada). All serum samples collected were stored at −80 °C.

### 2.6. Sequence Analysis

For sequence analysis, PCR I [14] targeting partial NS1 sequence of EqPV-H was performed according to Meister et al. PCR products were visualized on a 2% agarose gel, excised, and purified using a Monarch^®^ DNA Gel Extraction Kit (New England Biolabs). Purified products were then sent for Sanger sequencing using the applicable PCR primers. Published sequences were downloaded from https://www.ncbi.nlm.nih.gov/nucleotide/, accessed on 17 April 2020. Highlighter plot analysis [24] displaying nucleotide exchanges in partial EqPV-H NS1 was generated using the following website: https://www.hiv.lanl.gov/content/sequence/HIGHLIGHT/highlighter_top.html, accessed on 17 April 2020. First, sequences were aligned with muscle in MEGA 7 and sorted by sampling time. For each sampling time a highlighter plot was generated, sorted by similarity to a reference sequence (accession number MK792434) from a previously published strain originating from Europe. Afterwards all sequences were sorted by similarity within each time point and a new highlighter plot for all sequences was generated in the sorted order. Nucleotides are labelled according to IUPAC code. Sequences shown in phylogenetic trees were aligned in MEGA X with the ClustalW algorithm. Identical sequences of horses in this study were eliminated before performing the maximum likelihood phylogeny with 500 bootstraps. Concurrently, general time reversible model was used and gamma distribution with invariant site was chosen to model evolutionary rate differences [25,26,27]. Position with gaps and missing data were deleted completely. Variance estimation analyses were conducted using the Maximum Composite Likelihood model [28]. This analysis involved 79 nucleotide sequences. All ambiguous positions were removed for each sequence pair (pairwise deletion option). There was a total of 548 positions in the final dataset. Evolutionary analyses were conducted in MEGA X [26].

### 2.7. Illustrations

Graphs were plotted in GraphPad Prism (GraphPad software), and illustrations were generated with Adobe Illustrator.

## 3. Results

### 3.1. Evidence of Persistent and Chronic EqPV-H Infection in Horses from Germany

From October 2013 until 2018, veterinarians took horse serum samples for diagnostic purposes in a stable with 124 different horses over the years of this study. Of note, part of the same study cohort has previously been described by Pfaender et al. [11]. Not all horses could be sampled at every date as some had to be euthanized and new ones were introduced into the groups.

**Figure 1:**
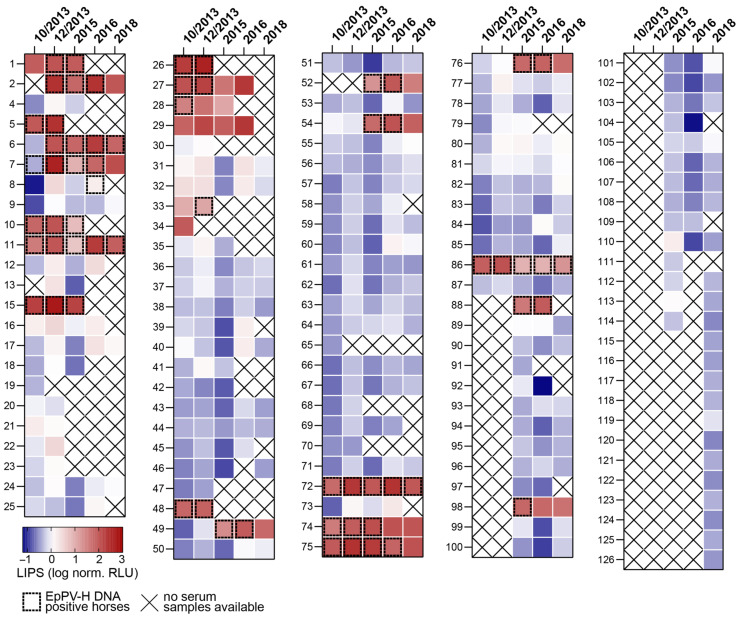
Study cohort overview. EqPV-H DNA was measured by qPCR, and antibodies against VP1 were determined by LIPS assay in horse serum taken at different dates. LIPS results of horse samples available for testing from October 2013 to 2018 are depicted as heatmaps. X indicates when serum samples were not available. Normalized RLU are shown as heat map. Antibodies measured in RLUs were normalized per plate as fold over threshold. Dotted squares indicate samples that were either tested positive by qPCR or deemed positive by detection of virus specific amplification on agarose gel.

Retrospectively, we tested all samples for EqPV-H by qPCR and LIPS assay for either the presence of viral DNA or anti-VP1 antibodies in 2019 (Figure 1). Most of the horses (62/82) were negative for both EqPV-H DNA and anti-VP1 antibodies in October 2013; however, EqPV-H DNA was present in horse 5, 7, 10, 11, 15, 26–28, 48, 72, 74, 75, and 86. We further observed different courses of infection among horses as exemplified by horses 6, 7, 11, 72 and 76.

Horse 6 was negative for EqPV-H DNA and anti-VP1 antibodies in October 2013 but tested positive for both in December 2013 and stayed EqPV-H positive for another 4 years as determined by either qPCR or visualization on agarose gel. Concurrently, anti-VP1 antibody counts peaked with ~191 fold-over the assay threshold in 2016 and decreased to ~65 fold-over by 2018.

In contrast, horse 7 was already infected with EqPV-H in October 2013 but did not develop antibodies against VP1 yet. VP1 antibodies were first detectable in December 2013 and remained detectable until 2018, while viral DNA was present until 2016. Detectable VP1 antibody counts decreased in 2015 to ~8 fold-over and steadily increased again until 2018 (~120 fold-over). No viral DNA was detected in 2018 by either qPCR or on agarose gel, potentially indicating clearance of infection five years after first EqPV-H DNA positive qPCR results.

Horse 11 has been persistently infected with EqPV-H over 5 years. Notably, VP1 antibody counts decreased in 2015 to ~5-fold from the previous year (~97 fold-over) and increased to ~198-fold two years later, in 2015. Of note, horse 11 has been tested negative for EqPV-H DNA in December 2013 and 2015 by qPCR; however, PCR product could be visualized, excised, and purified from the agarose gel, indicating that the infection might have persisted below LOD of qPCR.

Analysis of serum samples collected from horse 72 between 2013 and 2018, revealed the presence of EqPV-H DNA and VP1 antibody counts fluctuating between ~56 and 245-fold over threshold over five years, suggesting a chronic infection and a lack of ability to clear viral infection.

Horse 76 tested EqPV-H positive and seropositive for VP1 in 2015. Antibody counts continued to increase until 2016 with a peak at ~67 fold-over. By 2018, this horse was nonviremic and seropositive, potentially indicating clearance of EqPV-H viremia within two years after infection.

Overall, most horses in this cohort were negative for EqPV-H DNA and antibodies. However, some horses identified with EqPV-H infection exhibited prolonged viremia for at least two consecutive time-points. Horses that had no detectable EqPV-H-DNA in the serum in 2018 after being DNA positive for years hint at a possible clearance of infection.

### 3.2. Pathology of Chronic Horses

Histopathological analysis was performed to check for signs of hepatitis. Therefore, liver-biopsies of horses 6, 11, and 72 were collected in parallel to serum samples in 2019 and tested for the presence of viral DNA by qPCR (Table 2).

**Figure 2:**
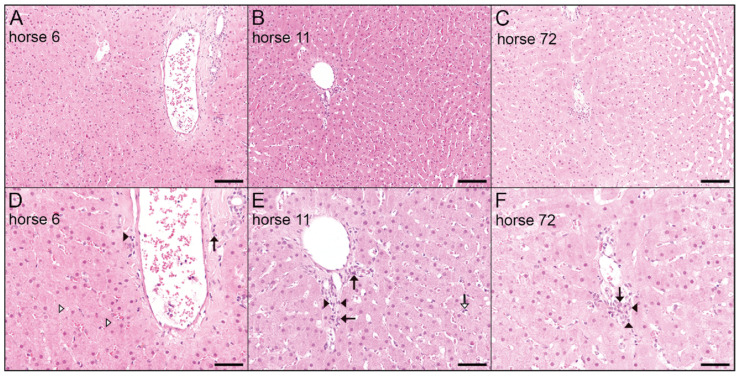
Histological results from biopsies of chronically EqPV-H-infected horses. (**A**,**D**) HE staining of liver biopsy samples of horse 6 with mild multifocal hepatocellular lipidosis (white arrowheads) and mild periportal infiltration with lymphocytes (black arrowhead) and macrophages (black arrow). (**B**,**E**) Mild periportal infiltration with lymphocytes (black arrowheads) and macrophages (black arrows) and a minimal, randomly distributed, suppurative inflammation (white arrow) within the liver of horse 11, HE. (**C**,**F**) Mild periportal infiltration with lymphocytes (black arrowheads) and macrophages (black arrow) within the liver of horse 72, HE. Images were taken at 10x magnification, 100 µm scale bar (**A**–**C**) and 20× magnification, 50 µm scale bar (**D**–**F**).

Additionally, liver-biopsies were fixed and HE stained. All biopsies displayed a similar morphological appearance. The regularly structured hepatic tissue showed a mild, multifocal, mostly periportally accentuated infiltration with lymphocytes and macrophages in all liver biopsies (Figure 2). Furthermore, in one biopsy, a mild multifocal (horse 6, white arrowheads) hepatocellular lipidosis was present. Notably, in horse 6, EqPV-H DNA was detectable in serum and liver biopsies. Additionally, multifocal hepatocytes and Kupffer cells contain low amounts of a cytoplasmic yellow-brownish pigment. In horse 11, a minimal, randomly distributed, suppurative inflammation was evident within the liver (white arrow). However, no EqPV-H DNA was detected in liver biopsy samples, and EqPV-H DNA in serum samples was below LOQ. In contrast, observation of infiltration with lymphocytes and macrophages in liver biopsy of horse 72 was accompanied by detection of serum titers of 1.6 × 10^4^ copies/mL and 2.3 × 10^3^ copies/µg DNA in serum and biopsy samples, respectively. Nevertheless, liver biopsies did not show signs of acute hepatic necrosis and scattered vacuolated hepatocytes.

### 3.3. Biochemical Analysis of Liver Enzymes in Viremic and Non-Viremic Horses

**Figure 3:**
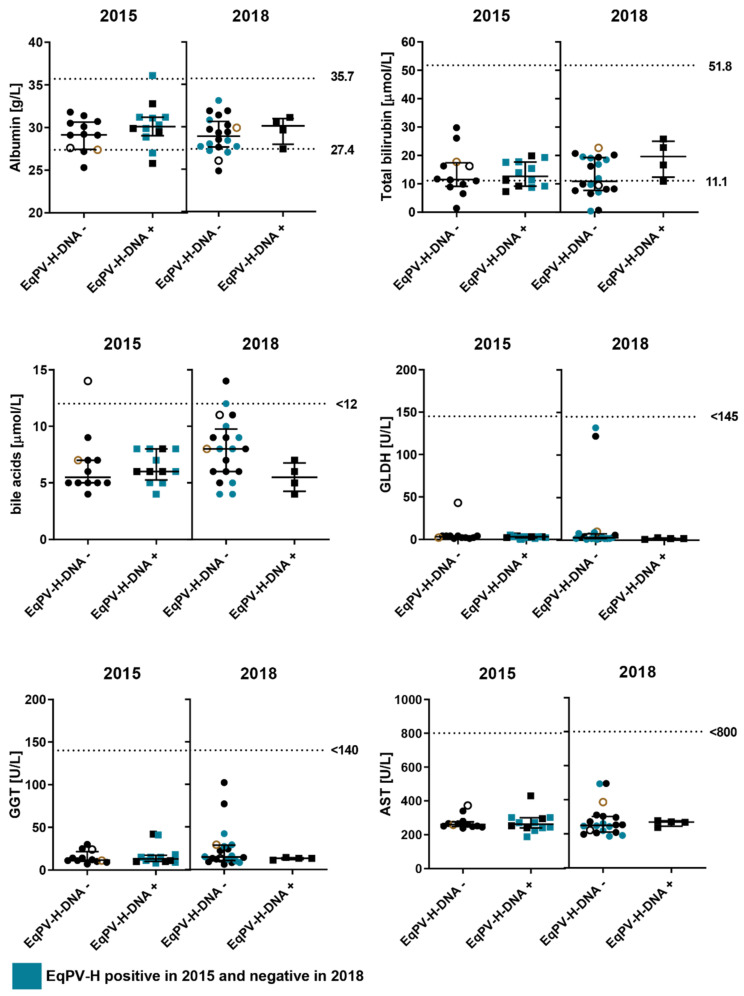
Liver specific biochemical analytes. Serum samples of 24 horses were used to determine their liver specific analytes in 2015 and 2018. Two groups divide horses depending on their qPCR result for EqPV-H. Horses are highlighted in blue if their qPCR result changed from samples taken in 2015 and 2018. Six analytes were measured and are shown: Albumin in grams/Liter (g/L) reference ranges between 27.4–35.7 g/L, total bilirubin in µmol/L; reference ranges between 11.1–51.8 µmol/L, bile acids in µmol/L; reference range is <12 µmol/L, glutamate dehydrogenase (GLDH) in units/Liter (U/L); reference range is <145, gamma-glutamyltransferase (GGT) in U/L; reference range is <140 and aspartate aminotransferase in U/L reference range is <800. Dotted line in each graph represents the corresponding standard cut-offs. Central tendency and variation are presented as median and interquartile ranges. Brown and black cycles indicate hepacivirus positive horses 56 and 61, respectively. Performing a 2-way ANOVA followed by Šídák’s multiple comparisons test, no significant differences between EqPV-H positive and negative samples were identified.

Liver compromise can lead to elevations in serum enzyme activities and an impaired albumin synthesis occurring in hepatic failure. Therefore, we determined biochemical analytes that could indicate hepatic disease in horse blood samples collected in 2015 and 2018. Blood samples were tested for serum liver enzymes (GGT, GLDH, AST), albumin, bilirubin, and bile acids (Figure 3). Overall, biochemical serum profile of viremic horses (EqPV-H+) remained unremarkable from 2015 and 2018. EqPV-H DNA negative samples from 2015 and 2018 were unobtrusive for liver disease as well, suggested by biochemical analytes values within normal range. However, minor decreases of albumin below the reference range as seen in this cohort are most likely non-specific and might be due to unrelated causes. Biochemical analytes in horses that were viremic (horse 7, 49, 52, 54, 74, 75, 76, 98) in 2015 and tested EqPV-H DNA negative in 2018 (blue dots) also remained consistent upon viral clearance. To properly evaluate biochemical and histopathology results, the possibility that horses were co-infected with other hepatic virus infections was ruled out by previous testing of horse serum samples for the presence of equine hepacivirus DNA and NS3 antibody by LIPS assay [11]. Horse 15 was EqPV-H and hepacivirus positive between October 2013 and 2015; however, samples were not sent to biochemical analysis or used for histopathology staining. In contrast, horse 56 and 61 have been negative for EqPV-H but tested positive for equine hepacivirus in 2015 (horse 61, Figure 3, black cycles) or 2018 (horse 56, Figure 3, brown cycles) and were used for biochemical analysis. Low albumin and bilirubin as well as high bile acid levels observed for horse 61 are most likely explained by hepacivirus infection.

In conclusion, viremic horses and horses with persistent viral EqPV-H infection did not show severe biochemical signs of hepatitis.

### 3.4. Cross-Species Detection of EqPV-H

**Figure 4:**
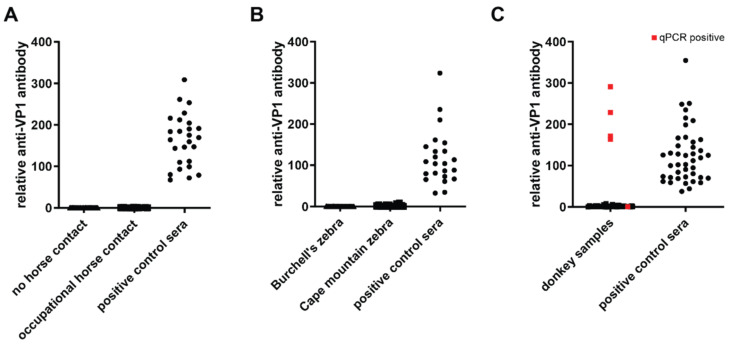
Detection of EqPV-H DNA and VP1 antibodies in serum samples from different species. qPCR and LIPS for EqPV-H were performed on serum samples of different species. Previously positive tested serum was used as positive control in LIPS assay. (**A**) Human sera were tested for the presence of anti-VP1 antibodies by LIPS. Study cohort was divided between people with or without horse contact as previously shown [11]. (**B**) Sera of two different zebra species were tested for the presence of anti-VP1 antibodies by LIPS. (**C**) Donkey serum samples (from Germany, Italy, and Bulgaria) were tested for the presence of EqPV-H DNA by qPCR and presence of anti-VP1 antibodies by LIPS. qPCR positive samples are colored in red.

The genus Equus, which includes all contemporary horses, donkeys, and zebras, originated approximately 4.5 million years ago [29]. Thus, the detection of EqPV-H homologues in equine sister species might aid decipher the evolutionary history and transmission patterns of EqPV-H. To investigate the possibility of cross-species infection of EqPV-H, we tested serum samples of humans with or without occupational horse contact [11] and donkeys for antibodies against EqPV-H capsid protein VP1 by LIPS assay (Figure 4). In addition, 65 Burchell’s and 231 Cape mountain zebra serum samples collected across South Africa between 2009–2017 were analyzed for viral DNA and presence of antibodies. Human samples of individuals with and without horse contact tested negative for both EqPV-H DNA (data not shown) and anti-VP1 antibodies (Figure 4A). Similarly, serum samples of Burchell’s and Cape mountain zebras were LIPS assay negative for anti-VP1 antibodies (Figure 4B) and EqPV-H DNA (data not shown). Intriguingly, four out of 494 donkey serum samples were seropositive for VP1 antibodies and tested positive for EqPV-H DNA (Figure 4C), suggesting EqPV-H infections in donkey. In addition, one donkey sample was tested positive by qPCR but not by LIPS assay. Of note, all five EqPV-H DNA positive samples contained a low viral load that was under the limit of quantification of qPCR.

### 3.5. Sequence and Phylogenetic Analysis

To assess whether different virus subtypes are circulating within the cohort, we analyzed the viral sequences of partial NS1 as described previously [14].

**Figure 5:**
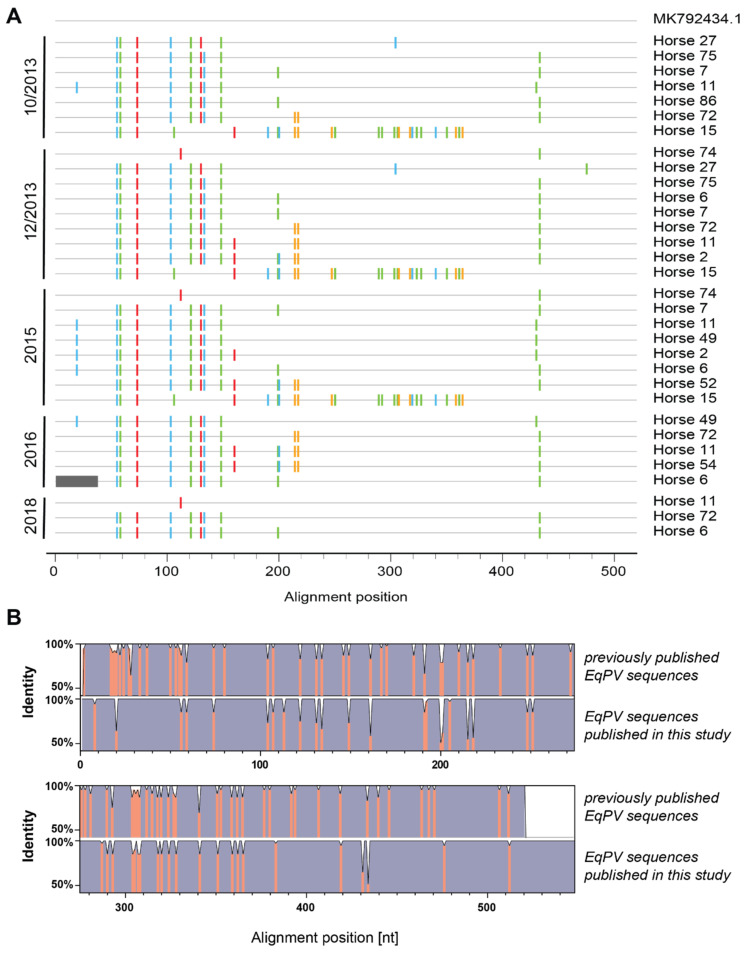
Sequence analysis of EqPV-H DNA positive samples. (**A**) Highlighter plot shows the nucleotide exchanges for different horses sorted by year and similarity to reference. MK792434.1, a previously published sequence from Europe, was used as reference. (**B**) Comparison of identity plots of previously published EqPV-H sequences and sequences from this study. Identity scores of respective multiple sequence alignments were obtained with Geneious 2021.1 (https://www.geneious.com, accessed on 24 June 2021).

When comparing the sequences from this study to a previously published sequence originating from a horse in Europe (accession number MK792434.1), we identified three different viral strains from 2013 until 2015 (Figure 5A). Only two of these remained in 2016, and only the major one of the previous year’s remained in 2018. Furthermore, virus strains persisted between 2013 and 2018 as exemplified by sequence analysis of horse 6 and 15. Additionally, sequences obtained from the same horses across the years were almost identical, suggesting a lack of intra-host mutations in these animals. Of note, although horse 11 was EqPV-H DNA negative in December 2013 and 2015 as determined by qPCR, we picked up sequences for horse 11 when visualizing and excising NS1 PCR product on an agarose gel and sending purified products for sequencing.

Comparing partial NS1 multiple sequence alignments (MSA) of previously published EqPV-H sequences and sequences identified in this study revealed similar identity scores within and between each MSA (Figure 5B). Only the 5′ end of the NS1 was more heterogenous in the previously published sequences. While estimations of the average evolutionary divergence over sequence pairs within previously published sequences and sequences found in horses in this study yielded 0.01607 ± 0.00194 and 0.01575 ± 0.00304 base substitutions per site, respectively, the average evolutionary divergence over sequence pairs between groups resulted in 0.0170 ± 0.0028 base substitutions per site. Therefore, the overall sequence similarity and nucleotide conservation was higher within this study cohort.

**Figure 6:**
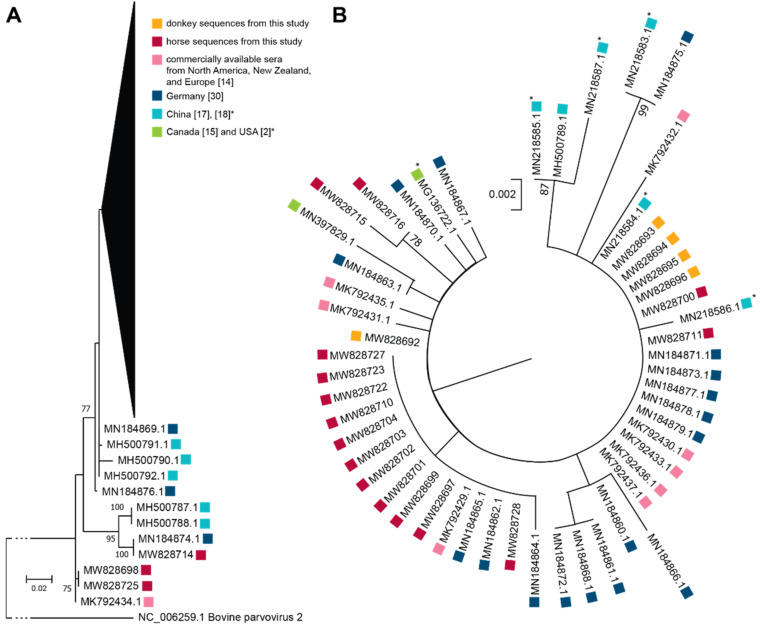
Molecular Phylogenetic analysis by Maximum Likelihood method. Maximum likelihood phylogenetic trees are drawn to scale with branch lengths measured in the number of substitutions per site. All positions containing gaps and missing data were eliminated. Evolutionary analyses were conducted in MEGA X [26] Bootstrap values >70% are shown. Red squares = sequences obtained in this study, rose squares = sequences obtained from commercially available serum pools [14], dark blue squares = sequences obtained from subclinical horses in Germany [30], bright blue squares = sequences from China [17] (with asterisks [18]), yellow squares = sequences obtained from horses in North America (Canada [15] (without asterisk) and USA [2] (with asterisk)). (**A**) The analysis involved 66 nucleotide sequences with 456 positions in the final dataset. We included the bovine parvovirus 2 reference strain as outgroup. (**B**) Sequences that belong to the collapsed part of the phylogenetic tree in A were reanalyzed including MN184869.1 as outgroup. The analysis involved 54 nucleotide sequences with 462 positions in the final dataset.

To determine the phylogenetic relationship between EqPV-H sequences from donkeys and other worldwide available EqPV-H horse serum samples and sera from this study, we performed phylogenetic analysis using the maximum likelihood method and general time reversible model. Therefore, sequences received from horse sera originating from North America (Canada [15] and USA [2]; green squares); Germany (dark blue squares) [30]; China (bright blue squares) [17,18]; and commercial horse sera from USA, New Zealand, Italy, France, Canada, and Europe (rose squares) [14] were compared to horse (red squares) and donkey (orange square) sequences identified in this study (Figure 6). Of note, we included bovine parvovirus 2 sequence as outgroup. Additionally, horse and donkey sequences from this study have been submitted to the National Center for Biotechnology Information (NCBI) database (Table 3).

As depicted in Figure 6, horse and donkey EqPV-H sequences obtained in this study clustered together with the already published sequences from horses in Europe, North America, New Zealand, and China. Obtained NS1 sequences were highly similar, thus pointing to a high conservation and low genetic variability between worldwide circulating EqPV-H strains and the horse and donkey strains from this study.

## 4. Discussion

In this longitudinal study, we first examined the persistence of EqPV-H infections in an isolated study cohort from Germany between 2013 and 2018. Based on qPCR and LIPS results, we identified chronic EqPV-H infections lasting up to five years in horses without severe clinical signs of hepatic disease. Histologic assessment of three infected horses showed non-specific abnormalities not attributable to reported acute liver necrosis seen with Theiler’s disease. Our findings are not uncommon in adult horses and cannot clearly be linked to the EqPV-H infection. In addition, whether EqPV-H was viable in liver samples could not be determined in this study.

The lack of biochemical evidence for liver disease—indicated by overall normal levels of liver enzymes, including GGT, GLDH and AST, as well as albumin, bilirubin, and bile acid levels—in EqPV-H DNA positive horses, has previously been demonstrated in studies conducted in subclinical EqPV-H positive horses. These studies have reported the presence of EqPV-H in healthy horses from cohorts in USA [2], China [17], Germany [30], and Austria [31] with prevalence rates of 13% (13/100), 11.9% (17/143), 7.14% (23/392), and 8.9% (23/259), respectively. In comparison, the percentage of viremic horses in this study varied between 5% (4/80 in 2018) and ~19.8% (16/81 in December 2013) from 2013 to 2018. Notably, factors determining why some horses develop hepatitis upon EqPV-H infection have yet to be determined. Regarding the biochemical analysis, we want to point out that reference ranges are well out of keeping with reference ranges at other labs using the same equipment.

Furthermore, we observed low genetic variability and high conservation between sequences from this study and previously published sequences from USA [2,14], Canada [15], China [17,18], Europe [14,30], and New Zealand [14]. Importantly, high similarity and conservation was also observed within sequences obtained from samples over the course of several years (e.g., horse 6 and 15). In line with these results, recent phylogenetic studies of worldwide circulating EqPV-H viruses have also described very low levels of genetic diversity of the NS1 sequence within their respective study cohorts and in comparison with sequences from other regions [14,15,17,30]. Interestingly, Baird et al. [15] have described a case about a sequenced serum sample of a horse that died of TD in 2016, eleven years after a documented outbreak in 2005, that had the same viral sequence of partial NS1 as samples from 2005. Although, the possibility that the horse had been infected prior to joining the farm in 2016 could not be excluded, they concluded that EqPV-H may have been circulating on this farm in Ontario Canada for almost a decade without causing outbreaks. Similarly, EqPV-H might have been circulating in the cohort from this study far longer than the here observed five years. At this point, we would also like to emphasize that the obtained results of high similarity and conservation as well as the lack of intra-host variability of NS1 sequences might differ when sequencing other genomic fragments. NS1 as a non-structural protein is probably under less immune selection—hence highly conserved—compared to VP1. Sequencing a less conserved region, such as VP1 or performing whole genome sequencing was not within the scope of this study; however, might have implications for interpreting conservation and intra-host variability of EqPV-H sequences in viremic horses. Interestingly, although genetic diversity was low, we identified virus sequences on different branches of the tree in one horse cohort.

The Animal and Plant Health Inspection Service (APHIS) from the U.S. Department of Agriculture (USDA) currently recommends administering USDA APHIS licensed and tested biologic blood products that have been PCR tested and confirmed negative for EqPV-H [32]. Thus, our observation, that prolonged low-level viremia can intermittently decrease below the detection limit of qPCR, as in the case of horse 11, might have important implications for the safety of using previously infected horses as blood donors. Likewise, prolonged viremia without clinical disease further emphasizes the diagnostic difficulty with ascribing hepatitis to parvoviral infection when viral load is low.

Lastly, we described EqPV-H viremia of donkeys with serum prevalence of ~1.0% (5/494), which is comparably low to serum prevalence found in horses. Additionally, we identified two different virus strains among four donkeys from Bulgaria. Further virological and serological surveys are needed to decipher the worldwide geographical distribution of EqPV-H infection in donkeys and possible transmission routes and mechanisms of cross-species transmission need further investigation. In addition, we have not examined levels of liver enzymes and the pathology of EqPV-H DNA positive donkeys in this study; hence, further research is needed to investigate putative signs of hepatitis in viremic donkeys.

In summary, our study documents the first reported cases of chronic EqPV-H infection in horses and the first observation of cross-species EqPV-H detection in donkeys.

## Figures and Tables

**Table 1:** Primer and probe sequences used for qPCR in this study.

Primer/Probe	Sequence
Forward primer	ATGCAGATGCTTTCCGACC
Reverse primer	GCCCCAGAAACATATGGAAA
Probe	[6-FAM]ACCGTAGCGGATTCGGGATCTGC[BHQ1a-6FAM]

**Table 2:** DNA copy numbers in liver biopsy samples from horses 6, 11, and 72.

Horse	EqPV-H DNA in Serum	EqPV-H DNA in Biopsy
6	6.4 × 10^3^ copies/mL	6.2 × 10^2^ copies/µg DNA
11	<2.5 × 10^3^ copies/mL	Not detected
72	1.6 × 10^4^ copies/mL	2.3 × 10^3^ copies/µg DNA

**Table 3:** Sequences from this study were submitted to the NCBI database. Each sample was assigned to the following accession numbers and sample ID refers to Figure 6.

Sample ID	Species	Country	NCBI Accession Number
BG128	donkey	Bulgaria	MW828692
BG147	donkey	Bulgaria	MW828693
BG153	donkey	Bulgaria	MW828694
BG155	donkey	Bulgaria	MW828695
I	donkey	Italy	MW828696
6_2018	horse	Germany	MW828697
11_2018	horse	Germany	MW828698
72_2018	horse	Germany	MW828699
2_2015	horse	Germany	MW828700
2_12/2013	horse	Germany	MW828701
6_2016	horse	Germany	MW828702
6_2015	horse	Germany	MW828703
6_12/2013	horse	Germany	MW828704
7_2015	horse	Germany	MW828705
7_12/2013	horse	Germany	MW828706
7_10/2013	horse	Germany	MW828707
11_2015	horse	Germany	MW828708
11_2016	horse	Germany	MW828709
11_12/2013	horse	Germany	MW828710
11_10/2013	horse	Germany	MW828711
15_2015	horse	Germany	MW828712
15_12/2013	horse	Germany	MW828713
15_10/2013	horse	Germany	MW828714
27_12/2013	horse	Germany	MW828715
27_10/2013	horse	Germany	MW828716
49_2015	horse	Germany	MW828717
49_2016	horse	Germany	MW828718
52_2015	horse	Germany	MW828719
54_2016	horse	Germany	MW828720
72_2016	horse	Germany	MW828721
72_12/2013	horse	Germany	MW828722
72_10/2013	horse	Germany	MW828723
74_2015	horse	Germany	MW828724
74_12/2013	horse	Germany	MW828725
75_12/2013	horse	Germany	MW828726
75_10/2013	horse	Germany	MW828727
86_10/2013	horse	Germany	MW828728

## Data Availability

Sequences generated in this study were uploaded to the NCBI nucleotide database and are available with the accession number MW828692-MW828728.
